# Selected Medicines Used in Iontophoresis

**DOI:** 10.3390/pharmaceutics10040204

**Published:** 2018-10-25

**Authors:** Tomasz M. Karpiński

**Affiliations:** Department of Medical Microbiology, Poznań University of Medical Sciences, Wieniawskiego 3, 61-712 Poznań, Poland; tkarpin@ump.edu.pl or tkarpin@interia.pl; Tel.: +48-61-854-61-38

**Keywords:** iontophoresis, drug delivery, dentistry, ophthalmology, local anaesthetics, non-steroid anti-inflammatory drugs, antibiotics, anticancer drugs

## Abstract

Iontophoresis is a non-invasive method of systemic and local drug delivery using an electric field. Iontophoresis enables diffusion of the selected drug via skin, mucosa, enamel, dentin, and other tissues. The amount of delivered therapeutic molecules is about 10–2000 times greater than conventional forms of delivery. Among other fields, this method is used in dentistry, ophthalmology, otorhinolaryngology, and dermatology. According to related literature, the most important drugs studied or administered by iontophoresis are: Local anesthetics, opioids, steroids, non-steroidal anti-inflammatory drugs, antibacterial drugs, antifungal drugs, antiviral drugs, anticancer drugs, fluorides, and vitamins. The present review covers current available data regarding the selected medicines used in iontophoresis. Furthermore, indications and conditions of iontophoresis application are reviewed.

## 1. Introduction

**Figure 1:**
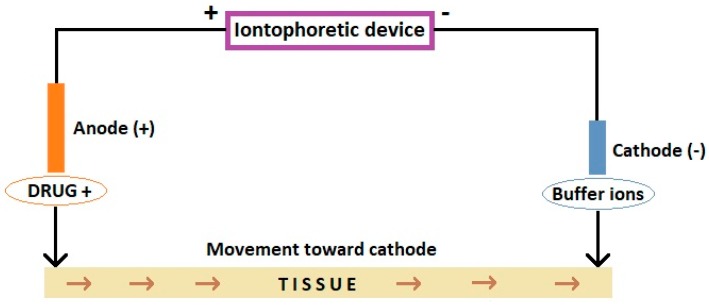
Scheme of iontophoretic device. Based on Dhote et al. [2], and Wanasathop and Li [7].

Iontophoresis is a type of electrotherapy, during which a drug is introduced deep into the tissues as a means of systemic and local drug application [1]. It is based on the principle that in a given electric field, positively charged drug ions (cations) are repelled by a positive electrode (anode) and are directed to the cathode (Figure 1). In turn, negative ions (anions, e.g., ketoprofen) being repelled by the negative electrode (cathode), follow the anode. The optimal molecules for use with iontophoresis are small and hydrophilic [2]. The penetration of the drug substance takes place through the skin, sweat glands and sebaceous glands, which enables diffusion to deeper layers of tissues, even 48 h after the procedure [3]. Iontophoresis allows delivery of about 10–2000 times more polar hydrophilic therapeutic molecules than typical application of a drug substance to the skin surface [4].

There are various factors affecting iontophoresis. Direct current is most commonly used, however in study of Zhu et al. alternating current (AC) iontophoresis showed better results than conventional constant current DC iontophoresis [5]. The drug used should be water-soluble, of low-dosage and susceptible to ionization. Smaller particles are more mobile. Increasing drug concentration results in greater drug delivery, but only to some extent. If buffer ions are present, they compete with the drug, reducing its delivery. The effect of iontophoresis also depends on the tissue on which the electrodes are applied (e.g., thickness, permeability, the presence of pores). In the skin, sweat glands are the most significant way the charges are carried [6].

The iontophoresis process uses two types of voltage supply—direct current and alternating current. The most commonly used method is direct current iontophoresis [8]. Among other fields, this method is used in dentistry, ophthalmology, otorhinolaryngology, and dermatology for the administration of local anesthetics, non-steroidal anti-inflammatory drugs, antibiotics, anticancer drugs, hormones, as well as other substances [8,9,10]. 

Some of the outstanding features of iontophoresis are its safety, high transdermal efficiency, and simplicity of application [11]. In contrast to other techniques, iontophoresis acts more on the molecules of medicine and less so on the skin [12]. However, direct current iontophoresis can cause side effects, such as burns, redness and skin irritation [8,13]. One significant problem of this method is that only potent molecules can be delivered [14].

Contraindications associated with the action of the galvanic current, as well as allergic drug reactions are:

- Superficial and deep sensory disorders;
- Should not be used in pregnancy;
- Should not be used during lactation;
- Hypersensitivity to the active substance [4,15,16].

The present review summarizes information about iontophoresis of the selected medicines from the following groups: Local anesthetics, opioids, steroids, non-steroidal anti-inflammatory drugs, antibacterial drugs, antifungal drugs, antiviral drugs, anticancer drugs, fluorides, and vitamins (Table 1). 

## 2. Local Anesthetics

Local anesthetics are drugs that cause a reversible absence of pain sensation [79]. Smitayothin et al. presented the anesthetized effect of iontophoresis of lignocaine with epinephrine on dentine for cavity preparation. The cavities of 56 molar teeth were filled with 20% lidocaine with 0.1% epinephrine. Iontophoretic anodal at 200 mA current was applied for 2 min. If the dentine was not anaesthetized, the treatment was repeated up to six times. Time of iontophoresis duration required to anaesthetize the dentine varied from: Two min for 7 teeth, 4 min for 17 teeth, 6 min for 14 teeth, 8 min for 4 teeth and up to 10 min for seven teeth. Not all studied teeth were anaesthetized. In 7 of the treated teeth, no effect was observed after 14 min of iontophoresis. Ultimately, 87.5% of the studied molars with caries were anesthetized by iontophoretic delivery of lignocaine with epinephrine [56]. Using lignocaine plus epinephrine solution iontophoresis on teeth caries immediately inhibits the pain and continues for at least 40 min [57]. In comparison, a locally applied solution of 50% lignocaine without iontophoresis produces anesthesia for a maximum of 10 min and after a latent period for up to 30 min [80].

The anesthetic effect of lidocaine is significantly increased after adding epinephrine and is dose-related. A concentration of epinephrine higher than 1:160,000 enhances and prolongs the anesthetic effect during the iontophoresis of lidocaine [48]. It was found that 2% lidocaine with epinephrine can be delivered up to 5 mm below the surface of the skin [55].

Inoue et al. presented that lidocaine is delivered in iontophoresis more rapidly with direct current than with alternating current. Moreover, ions are transported faster when voltage is switched from direct current to alternating current. They found that iontophoresis in combination with direct current and alternating current enabled highly efficient drug delivery [8].

It was also shown that lidocaine hydrochloride can pass through enamel/dentine by using alternating current iontophoresis [51]. This method may avoid the numbness observed when the anesthetic is administered with a syringe [81]. The permeability of enamel/dentine may function as a novel drug delivery system. Using this system of iontophoresis, antibiotics and nonsteroidal anti-inflammatory drugs (NSAIDs) can be administered in order to alleviate inflammation and pulpal pain or dentine hypersensitivity following bleaching or acid etching of enamel [51,82]. Saliba et al. demonstrated that high amplitude/short duration or low amplitude/long duration of lidocaine iontophoresis does not affect the anesthetic level when the same dosage is applied [83].

## 3. Opioids

The fentanyl HCl iontophoretic transdermal system (fentanyl ITS) has been approved in the US and Europe. This system ensures patient-controlled analgesia in acute and moderate-to-severe postoperative pain [84]. Fentanyl ITS is considered an equivalent method of pain control comparative to standard intravenous delivery of morphine [37]. The described system also decreases staff assistance time required for helping patients and accelerates the anesthetic effect [85].

## 4. Steroids

Iontophoresis is used as a non-invasive method for administration of dexamethasone into the eye for the treatment of uveitis and other inflammatory eye conditions. It was demonstrated that iontophoresis enhances penetration and distribution of a combination of dexamethasone and dendrimers into the cornea [33]. A study with patients suffering from dry eye revealed that receiving dexamethasone phosphate (EGP-437) solution via ocular iontophoresis led to reducing unwanted signs and symptoms [86]. Iontophoretic application of dexamethasone phosphate (EGP-437) into ocular tissues can also be used for therapy of anterior uveitis [87] and ocular inflammation post cataract surgery [88].

Some studies have also considered the use of dexamethasone in iontophoresis for treating disorders of temporomandibular joints. Dexamethasone/dexamethasone phosphate can be delivered to depths of up to 12 mm [89]. For disorders of temporomandibular joints, dexamethasone iontophoresis improves function, including motion and affects decreasing pain [32,90].

## 5. Non-Steroidal Anti-Inflammatory Drugs

Non-steroidal anti-inflammatory drugs (NSAIDs) have anti-inflammatory, analgesic and antipyretic properties. Data from literature indicate that the use of NSAIDs in percutaneous iontophoresis of temporomandibular joints relieves pain in rheumatoid arthritis, joint injuries and for patients with masticatory dysfunction [91,92]. The application is also used in post-traumatic conditions (e.g., dislocation of the temporomandibular joints), overload conditions (e.g., in the course of bruxism), chronic inflammatory and degenerative-inflammatory processes, neuralgias, lockjaw, tooth hyperalgesia and postoperative pain [93]. NSAIDs used in iontophoresis are applied omitting oral administration and thus save the gastrointestinal tract, especially for patients with diseases and dysfunctions of the digestive system [3].

Ketoprofen given percutaneously penetrates well into temporomandibular joints. It was found that the higher the activity of the synovium inflammatory process, the better the penetration of the drug into the joint cavity [94]. The cationic ester prodrug of ketoprofen highly reduced plasma protein binding. Anodal iontophoresis of the cationic ester prodrug of ketoprofen is characterized by enhanced delivery efficiency and increased drug retention in the skin and has potential in topical therapy of musculoskeletal diseases [47].

Other NSAIDs used in iontophoresis are aspirin, ibuprofen, and indomethacin. The studied NSAIDs administered by iontophoresis showed a higher anti-inflammatory effect in rats than drugs administered without iontophoresis. Iontophoresis significantly improved penetration of aspirin, ibuprofen, and indomethacin into the hypodermis, dermis and epidermis [11]. In the case of celecoxib using iontophoresis enhanced transdermal flux through rat skin resulted in almost twice as much drug transport in comparison to passive diffusion. Iontophoretic application of celecoxib can be used in the treatment of osteoarthritis, acute pain, joint inflammation and joint injuries [26]. 

In studies on humans, iontophoretic delivery of diclofenac demonstrated measured plasma concentrations of this drug in 75% of participants. However, in the group without iontophoresis only in 25% of the subjects was diclofenac detected in their plasma. The authors suggested that iontophoresis failed to achieve more effective local concentrations of diclofenac [35]. Arunkumar et al. used terpenes (geraniol, l-menthol and thymol) as iontophoretic efficiency enhancers. They presented the effect of terpenes on iontophoresis of diclofenac. Geraniol and l-menthol enhanced the iontophoretic flux, decreased the percent of inflammation, and enabled safer iontophoresis of diclofenac [95].

Presence of dental caries may inhibit drug delivery from dentine to the pulp. Puapichartdumrong et al. investigated the role of iontophoresis in drug delivery through human intact dentine and dentine affected with caries. The diffusion of metronidazole, sodium salicylate, and naproxen sodium was significantly less through dentine with caries than those without a cavity. Simultaneously, iontophoresis enhanced the delivery of drugs [58].

## 6. Antibacterial Drugs

Iontophoresis can be used as a method of disinfection against biofilm in teeth root canals. Gergova et al. showed that potassium iodide iontophoresis was most effective against biofilm caused by Gram-positive cocci. A very good bactericidal effect was also obtained by iontophoresis with Cupral (Cu). The authors emphasize that iontophoretic disinfection is a non-invasive and low-cost method [29].

Some studies on animals have shown the influence of antibiotics when used in ocular iontophoresis. Choi and Lee presented that transscleral and transcorneal iontophoresis (0.5 mA for 5 min) is suitable for application of vancomycin into the aqueous and vitreous humor and the cornea of rabbit eyes [77]. Research, has shown that transscleral transport of vancomycin does not increase linearly with either an increase of current density or antibiotic concentration [78]. Rabbits were also used to study transcorneal iontophoresis for delivery of ciprofloxacin. The authors described this method as being useful for ciprofloxacin delivery into the aqueous humor for the treatment of intraocular infections [28]. Iontophoresis of hydrogel containing gentamicin can be used for eye infections. Rabbits were used as subjects to describe the potential clinical effect of gentamicin-loaded hydrogels in treating corneal infections caused by *Pseudomonas aeruginosa* [44]. Iontophoresis increased also concentration of amikacin in rabbit eye and skin [22,23].

Iontophoresis is a promising method for fast application of amoxicillin and cefuroxime into the skin. In the case of amoxicillin, therapeutic concentrations in the skin were detected immediately after the application and remained for at least two h. For cefuroxime, therapeutic skin concentrations were obtained only at higher current densities [25]. In studies of Puapichartdumrong et al. diffusion of metronidazole, drug used against anaerobic bacteria, was significantly influenced by iontophoresis [58].

Silver also possesses antibacterial activity. Iontophoresis with a silver-polymer-based surface system caused a high antibacterial effect against Gram-negative bacteria (*Escherichia coli*, and *Pseudomonas aeruginosa*) and low effect against Gram-positive bacteria (*Staphylococcus aureus* and *Enterococcus faecalis*) [64].

Application of anodal iontophoresis using silk fibroin and neurotensin caused the release of high neurotensin concentrations in a short period. This type iontophoresis had an additional bacteriostatic effect against Gram-positive *Staphylococcus aureus* and *S. epidermidis*, without causing toxicity to fibroblasts [63]. Whereas, anodal iontophoresis at pH 4.0 increased transport and accumulation of amikacin used topically for the treatment of ocular infections [23].

## 7. Antifungal Drugs

Grossman and Lee described that ketoconazole concentrations in aqueous humor and cornea of the rabbit eye were significantly higher after transscleral and transcorneal iontophoresis [46]. Sachdeva et al. applied terbinafine hydrochloride to the skin using anodal iontophoresis. It was observed that iontophoresis delivered higher drug levels to the deeper skin as compared to control subjects. The drug was detectable in the skin for at least two days following iontophoretic treatment [76]. Terbinafine provides fungicidal activity against dermatophytes (*Trichophyton*, *Microsporum*, and *Epidermophyton*), molds and certain dimorphic fungi. When used against fungi of the genera *Candida* and *Malassezia* it either acts as fungicidal or fungistatic, depending on the species [96].

## 8. Antiviral Drugs

Siddoju et al. described the use of iontophoresis for improved topical delivery of acyclovir. Acyclovir is used to treat Herpes simplex and Varicella-zoster virus infections. Iontophoresis was performed for 10 min on rats using a 5% acyclovir gel. The method resulted in high acyclovir levels in skin layers for up to 2–3 days [20]. Transscleral iontophoresis of acyclovir prodrugs (ACV-X, X = Arg, Gly and Trp) achieves a higher concentration and faster delivery rate of acyclovir. Iontophoresis of ACV-Gly for 5 min leads to a higher level of acyclovir than IC_50_ against HSV-1. This method is useful for the treatment of herpetic infections in the anterior and posterior segments of the eye [21].

## 9. Anti-Cancer Drugs

Local iontophoretic chemotherapy treatment at various stages of cancer can be an addition to surgery, radiation, and systemically administered chemotherapy. Local treatment leads to the reduction of drug side effects and is suggested for drugs which are too toxic when delivered systemically [97,98]. The iontophoretic application of anticancer agents can act transdermal, transpapillary, intravesical, transscleral, and peri-pancreatic [10].

Cisplatin and 5-fluorouracil are drugs used in the treatment of skin basal cell and squamous cell carcinoma. Iontophoretic application of 5-fluorouracil led to no clinical or histologic evidence of residual squamous cell carcinoma three months after the last treatment in 25 of 26 patients [99]. The effect of local administration of 5-fluorouracil combined with immunoliposomes using iontophoresis against squamous cell carcinoma was also described [39]. The cisplatin iontophoresis was used successfully in a patient with basal cell carcinoma. The effectiveness of the treatment was confirmed by biopsies, which revealed no evidence of basal cell carcinoma [100]. 

Chemotherapeutic agents to treat head and neck cancers can be delivered using buccal iontophoresis. Cathodal iontophoresis of 5-fluorouracil and leucovorin increased the mucosal deposition of both drugs. An 8-fold enhancement of deposition for 5-fluorouracil and a 3-fold increase for leucovorin was observed [38].

Iontophoretic cancer treatment was used to apply nanocomplex STAT3 siRNA (a small interference RNA) with success in preclinical studies [101]. STAT3 (signal transducer and activator of transcription 3) is an oncogenic transcription factor which is activated in multiple cancer types, including skin cancer [102]. Topical iontophoresis of curcumin with STAT3 siRNA complex enhanced skin penetration of the nanocomplex and can be developed for skin cancer treatment [30]. Iontophoretic anodal application of STAT3 siRNA complex and imatinib mesylate had a suppressive effect on melanoma cancer [75].

Another possibility of iontophoretic cancer treatment is for administering anti-cancer vaccines. Toyoda et al. studied nanogels containing gp-100 peptide KVPRNQDWL as a potential component of anti-cancer vaccines against melanoma in mice. The iontophoresis improved transport of the nanogels into the skin, as well as accumulation of Langerhans cells. Finally, melanoma growth in mice was significantly suppressed by this method [45].

Iontophoresis for anticancer therapy is also used in connection with other methods. One example is a combination of direct-current pulsed iontophoresis along with photodynamic therapy. These two combined methods can be used for application of 5-aminolevulinic acid for skin cancer. Patients with actinic keratosis who were treated with iontophoresis and photodynamic treatment had complete recovery [24].

## 10. Fluorides

Fluoride iontophoresis can be used for reduction of dentinal hypersensitivity and remineralization of enamel. Patients affected by dentinal hypersensitivity have symptoms reacting to hot, cold, chilled, acidic or sweet liquids and food. Fluoride ions react with calcium ions forming acid-resistant calcium fluoride which blocks dentinal tubules [68]. It was demonstrated that iontophoresis with 2% sodium fluoride (NaF) solution is more effective in reduction of hypersensitivity than local application of 2% NaF or HEMA-G iontophoresis. Measurements at 3-month intervals of administering 2% NaF iontophoresis indicated the best long-lasting effect [70]. A similar effect was observed Singal et al. The Authors compared reduction of dentinal hypersensitivity after using 2% NaF iontophoresis and an aqueous solution of HEMA-G. NaF iontophoresis had a more significant effect in the 1- and 3-month intervals than HEMA-G [67]. 

Using in vitro studies, fluoride iontophoresis was compared with the conventional application of fluoride. Bovine enamel samples were covered with 3 mL of fluoride (1.23% acidulated phosphate fluoride (APF) gel and 2% sodium fluoride (NaF) solution) for 4 min each 5 days. The iontophoresis study group was electrically charged for 4 min each five days. In this experiment, fluoride iontophoresis was not significantly better than conventional fluoride application regarding the remineralization effect [18].

In other studies, also based on bovine enamel, the most effective treatment was acidulated phosphate fluoride gel application, followed by 5% NaF varnish application and 2% NaF iontophoresis. In the NaF iontophoresis group the fluoride uptake was higher, and cavity depth was smaller, in comparison to the control group. However, NaF iontophoresis had a significantly lower effect on the remineralization than that of acidulated phosphate fluoride gel [69].

## 11. Vitamins

Iontophoresis with dextran-free 0.1% riboflavin-5-phosphate (Vitamin B2) solution was used in a randomized study of patients with progressive keratoconus. Iontophoresis was delivered along with irradiating the cornea with a 10 mW/cm^2^ ultraviolet A for 9 min. One year following iontophoresis significant visual and refractive improvements were reported. Simultaneously, no complications occurred in this patient group [62]. Other studies have shown inhibition of keratoconus progression within 24 months, resulting in a significant improvement in visual and topographic parameters. Riboflavin (0.1%) was used in iontophoresis-assisted corneal crosslinking [61]. Application of riboflavin in corneal stroma by iontophoresis for halting keratoconus progression was also suggested by Mencucci et al. [59].

An animal model was tested using riboflavin/ultraviolet-A iontophoresis-assisted cross-linking procedure for the treatment of myopia. Riboflavin (0.1%) was used as a photosensitizer and the duration of iontophoresis was 10 min. This combination of methods was deemed effective and safe to control the pathologic process of myopia [60].

## 12. Summary

To summarize, iontophoresis is a valuable, non-invasive method, which can be used for topical treatment of pain, inflammation, infections, and cancers. Some medicines, e.g., lignocaine or sodium fluoride are already administered by iontophoresis to patients. Other medicines are under in vitro investigations or undergoing animal studies and have shown potential for applying them for delivery of different medicines. Further research should be performed especially on the effectiveness of delivering non-ionic drugs using this method.

## Figures and Tables

**Table 1:** Summary of selected studies concerning iontophoretic application of drugs.

Drug	Iontophoresis Conditions	Tissue	References
Acetylsalicylic acid	5 mA/cm^2^, 10 min	In vivo rabbit eye	[17]
Acetylsalicylic acid (Aspirin)	0.06 mA, 10 min	Rat skin	[11]
Acidulated phosphate fluoride	0.4 mA, 4 min	Bovine enamel	[18]
Acidulated phosphate fluoride	0.2 mA, 4 min	Human teeth	[19]
Aciclovir	0.2 mA/cm^2^, 10 min	In vivo rat skin	[20]
Aciclovir	0.5 mA/cm^2^, 5 min	Porcine eye	[21]
Aciclovir prodrugs	0.5 mA/cm^2^, 5 min	Porcine eye	[21]
Amikacin	3.7–7.4 mA/cm^2^, 20 min	In vivo rabbit eye	[22]
Amikacin sulphate	0.5 mA/cm^2^, 2 h	Rabbit skin	[23]
5-Aminolevulinic acid	1–2 mA, 10 min	In vivo human skin	[24]
Amoxicillin	100, 200, 300 µA/cm^2^	In vivo rabbit skin	[25]
Cefuroxime	100, 200, 300 µA/cm^2^	In vivo rabbit skin	[25]
Celecoxib	0.1–0.5 mA/cm^2^, 25–300 min	Rat skin	[26]
Ciprofloxacin	0.8 mA, 10 min	In vivo rabbit cornea	[27]
Ciprofloxacin hydrochloride	6.25 mA/cm^2^, 5 min	Porcine cornea and whole eye	[28]
Cupral	1.5 mA, 10 min	Human teeth root canals	[29]
Curcumin with STAT3 siRNA	0.47 mA/cm^2^, 4 h	Porcine skin	[30]
Dexamethasone	0.36 mA/cm^2^, 10 and 20 min	In vivo human skin	[31]
Dexamethasone	4 mA, 15–30 min	In vivo human temporomandibular joint	[32]
Dexamethasone	5.1 mA/cm^2^, 4 min	In vivo rabbit cornea	[33]
Dexamethasone	1 mA/cm^2^, 180 min	Porcine cornea	[33]
Dexamethasone	0.1 mA, 8 h	Bovine palate	[34]
Diclofenac	5 and 10 mA, 20 min	In vivo human muscle	[35]
Diclofenac sodium	0.5 mA/cm^2^, 8 h	Porcine skin	[36]
Ibuprofen	0.06 mA, 10 min	Rat skin	[11]
Indemethacin	0.06 mA, 10 min	Rat skin	[11]
Fentanyl hydrochloride	10 min	In vivo human skin	[37]
5-Fluorouracil	1 mA/cm^2^, 10 and 20 min	Bovine buccal mucosa	[38]
5-Fluorouracil	0.5 mA/cm^2^, 15 min and 6 h	Porcine skin	[39]
Gentamicin sulfate	2.5–5.1 mA/cm^2^, 1 and 2 min	In vivo rabbit cornea	[40]
Gentamicin sulfate	5.1 mA/cm^2^, 1 min	In vivo rabbit cornea	[41]
Gentamicin sulfate	0.51–3.1 mA/cm^2^, 1 min	In vivo rabbit cornea	[42]
Gentamicin sulfate	40 mA, 1, 5 and 10 min	Sheep and human bone	[43]
Gentamicin sulfate	0.8 and 2 mA/cm^2^, 1 min	In vivo rabbit cornea	[44]
Gp100 peptide	0.4 mA/cm^2^, 1 h	In vivo mouse melanoma	[45]
Ketoconazole	4–6 mA, 15 min	Rabbit eye	[46]
Ketoprofen cationic prodrug	0.2 mA, 6 h	Rat skin	[47]
Leucovorin	1 mA/cm^2^, 10 and 20 min	Bovine buccal mucosa	[38]
Lidocaine	1 mA, 10 min	In vivo human skin	[48]
Lidocaine	0.3 mA, 8 h	Porcine buccal mucosa	[49]
Lidocaine	0.3 mA/cm^2^, 8 h	Porcine buccal mucosa	[50]
Lidocaine	3 V, 1 kHz, 2–20 min	Human enamel/dentine	[51]
Lidocaine	1 mA/cm^2^, 1 h	Porcine buccal mucosa	[52]
Lidocaine	0.5 mA/cm^2^, 6 h	Porcine buccal mucosa	[53]
Lidocaine	0.5–5 mA/cm^2^ or 0.5–20 mA/cm^2^, 30 min	Rabbit cornea or rabbit conjunctiva	[54]
Lignocaine with epinephrine	1 mA, 10 min	In vivo human skin	[48]
Lignocaine with epinephrine	40 mA, 10.5 min	In vivo human skin	[55]
Lignocaine with epinephrine	0.2 mA, 2–14 min	Human dentine	[56]
Lignocaine with epinephrine	0.12 mA, 90 s	Human dentine	[57]
Metronidazole	0.05 mA, 10 min	Human dentine	[58]
Naproxen sodium	0.05 mA, 10 min	Human dentine	[58]
Potassium iodide	1.5 mA, 10 min	Human teeth root canals	[29]
Riboflavin	1 mA, 5 min + UVA irradiance	Human cornea	[59]
Riboflavin	10 min	In vivo rabbit eye	[60]
Riboflavin	1 mA, 5 min	In vivo human eye	[61]
Riboflavin-5-phosphate	1 mA, 5 min	In vivo human cornea	[62]
Silk fibroin with neurotensin	0.2 mA/cm^2^, 30 min	Macrophages	[63]
Silver-polymer-based surface	1.5–15 µA, 30 min	Bacterial strains	[64]
Sodium fluoride	0.5–0.6 mA, 30 s	Rat enamel	[65]
Sodium fluoride	10 mA, 2–3.3 min	Human enamel	[66]
Sodium fluoride	0.5 mA, 2 min	In vivo human teeth	[67]
Sodium fluoride	0.5 mA, 5 min	Human dentine	[68]
Sodium fluoride	0.4 mA, 4 min	Bovine enamel	[18]
Sodium fluoride	0.2 mA, 4 min	Bovine enamel	[69]
Sodium fluoride	0.5 mA, 2 min	In vivo human teeth	[70]
Sodium fluoride	0.2 mA, 4 min	Human teeth	[19]
Sodium fluoride	0.1 mA, 8 h	Bovine enamel	[71]
Sodium fluoride	0.1 mA, 8 h	Bovine palate	[34]
Sodium fluoride	0.3 mA, 4 min, for 5 days	Bovine enamel	[72]
Sodium fluoride	1.5 mA, 3 min	Human dentine	[73]
Sodium fluoride	0.5 mA, 3 min 0.5 mA, 5 min 0.5 mA, 10 min	Bovine enamel	[74]
Sodium salicylate	0.05 mA, 10 min	Human dentine	[58]
Sodium salicylate	0.1 mA, 8 h	Bovine palate	[34]
STAT3 siRNA with imatinib mesylate	0.5 mA/cm^2^, 2 h	In vivo mouse melanoma	[75]
Terbinafine hydrochloride	0.25 mA, 10, 15 and 20 min	In vivo rat skin	[76]
Vancomycin	0.5–3.5 mA, 10 min	Rabbit eye	[77]
Vancomycin	2.55–10.2 mA/cm^2^, 120 min	Rabbit sclera	[78]
